# Lead exposure sources and public health investigations for children with elevated blood lead in England, 2014 to 2022

**DOI:** 10.1371/journal.pone.0304866

**Published:** 2024-07-18

**Authors:** Mona Dave, Araceli Busby, Lena Al Shammari, Neelam Iqbal, Louise Coole, Helen Bagnall, Helen Crabbe

**Affiliations:** 1 UK Field Epidemiology Training Programme, UK Health Security Agency, London, United Kingdom; 2 Field Service Midlands, Regions Directorate, UK Health Security Agency, Birmingham, United Kingdom; 3 North East and North Central London Health Protection Team, UK Health Security Agency, London, United Kingdom; 4 Environmental Epidemiology Group, Radiation Chemicals and Environmental Hazards Directorate, UK Health Security Agency, Oxfordshire, United Kingdom; 5 Institute of Infection, Veterinary and Ecological Sciences, University of Liverpool, Liverpool, United Kingdom; Kermanshah University of Medical Sciences, ISLAMIC REPUBLIC OF IRAN

## Abstract

**Background:**

Lead exposure at any concentration can adversely impact health, with children being more vulnerable to its effects. In England, children with an elevated blood lead concentration (BLC) are reported to Health Protection Teams (HPTs) for public health investigation. A detailed review of these cases has not yet been conducted.

**Objectives:**

The objectives of this study were to describe the demographics, likely setting and sources of lead exposure, risk behaviours, public health investigations and outcomes for children aged <16 years with a BLC requiring public health action reported to HPTs between 2014–2022 in England.

**Methods:**

Data were collected via a lookback questionnaire and a live enhanced surveillance questionnaire. Data were deduplicated, cleaned and results summarised as numbers and percentages using R studio. A thematic analysis was conducted on qualitative responses to a question relating to problems experienced during case investigation.

**Results:**

There were 340 cases in our study: the majority were aged 1–4 years old (53%) and male (69%). Ethnicity data was poorly recorded. A higher than expected proportion (31%) lived in the most deprived areas. Pica (76%) and learning difficulties (60%) were often present. Cases were primarily exposed to lead in the domestic setting (92%) with paint (43%) and soil (29%) the most common exposures. Most cases lived in rented accommodation (63%), with a higher proportion in social rentals (48%) than privately rented (37%). Case investigations were resource intensive and poor stakeholder engagement/response was most frequently identified as challenging by HPTs.

**Conclusions:**

Lead exposure is harmful to children and requires public health and clinical management, which can be complex and challenging. Prevention of lead exposure in children should be the focus of intervention efforts. Outreach, engagement and preventative work should focus on both renters and homeowners. Collecting ethnicity data consistently may enable identification of more specific groups at increased risk of lead exposure in England.

## Introduction

### Public health importance of lead exposure

Lead is a non-essential element and exposure can cause adverse health impacts at any level [1, 2]. Lead exposure can result in symptoms such as abdominal pain, vomiting, loss of appetite, constipation, hearing loss, irritability and fatigue, developmental delay and learning difficulties [3]. Children are more vulnerable to the adverse effects of lead exposure as lead is likely to deposit and accumulate in their growing bones and organs [3].

Approximately 1 in 3 children–up to 800 million globally–have blood lead levels ≥0.24μmol/L (≥5μg/dL), at which concentration the World Health Organisation (WHO) [4] considers intervention is required. In the United States the intervention concentration has recently been lowered by the Centres for Disease Control and Prevention (US CDC) to ≥0.17μmol/L (≥3.5μg/dL) [5]. In England, the current blood lead concentration (BLC) which triggers public health intervention is ≥0.24μmol/L (≥5μg/dL) for both children and the developing foetus (pregnant women) and ≥0.48μmol/L (≥10μg/dL) for adults [6]. The intervention concentration for children was lowered from ≥0.48μmol/L (≥10μg/dL) to ≥0.24μmol/L (≥5μg/dL) from 5^th^ July 2021 [7] following a review published by Public Health England’s (PHE, now UK Health Security Agency (UKHSA)) Lead Intervention Concentration Working Group [1].

In the United Kingdom (UK), measures have been taken to reduce human exposure to lead though prohibiting the use of lead in petrol, paint, food cans and water pipes. However, historic circulation of lead-containing products and the importation of lead-containing products from outside of the UK still occurs [8].

There is no population level screening programme for lead poisoning in the UK. Screening for lead poisoning in asymptomatic children aged between 1 and 5 years is not currently recommended due to concerns relating to unknown prevalence of elevated blood lead concentrations (BLC) and complexity around testing and treatment of children [9]. Blood lead testing in the UK is therefore conducted on a case-by-case basis, often as part of generalised clinical investigations around pica behaviour (often associated with autism) or on clinical suspicion of lead poisoning. Due to the non-specific nature of symptoms, lead exposure can be missed or misdiagnosed. Blood tests for BLC in England are analysed by biochemical laboratories, predominantly at National Health Service (NHS) Supra-regional Assay Services (SAS) Trace Elements laboratories [10], or NHS Trust biochemical laboratory services [11].

### The Lead Exposure in Children Surveillance System (LEICSS)

UKHSA’s Lead Exposure in Children Surveillance System (LEICSS) is a national, passive surveillance system for children residing in England aged <16 years who have an elevated BLC requiring public health action. There are currently 14 participating laboratories which send notifications of elevated BLC at a concentration reportable for public health action (currently ≥0.24μmol/L (≥5μg/dL)) to LEICSS (6 NHS SAS Trace Elements laboratories and 8 other NHS and private laboratories) [11]. All surveillance reporting laboratories are UK Accreditation Service (UKAS) accredited [11].

LEICSS team administrators will then send these notifications on to regional health protection teams (HPTs) for follow up and action based on UKHSA’s national standard operating procedure (SOP) for case management. HPTs may also receive case notifications for both children and adults directly from laboratories, health professionals treating the case or the UK National Poisons Information Service (NPIS) [12]. LEICSS extracts all notifications to HPTs of elevated blood lead in children aged <16 years in England for surveillance purposes, summarising case demographics in LEICSS annual reports [13, 14]. Further information on the surveillance processes and BLC testing regime is available in these reports.

### Study rationale and aim

Whilst case demographics are described in LEICSS annual reports [13, 14], a detailed review of exposures, public health investigations and case outcomes for children with an elevated BLC reported to HPTs in England has not yet been conducted. Understanding these factors can give an insight to population level characteristics of exposures, risk factors and outcomes which helps to inform public health intervention, prioritise areas for prevention efforts and improve case management for child cases of elevated blood lead in England.

## Methods

This was a descriptive cohort study of the epidemiology of cases of elevated BLC aged <16 years, resident in England and notified to HPTs between 2014 and 2022.

### Data collection

For cases reported to HPTs between 1^st^ January 2014 and 4^th^ July 2021, a lookback questionnaire was sent to case managers to collect information on case demographics (age, sex, ethnicity), source of notification, household details, public health investigations (details of site visit, likely source and setting of lead exposure), pica, control measures implemented, outcomes and clinical care cases received. This required a manual review of the case records in the HPT case management system (HPZone). For cases reported to HPTs between 5^th^ July 2021 and 30^th^ September 2022 (end of data collection period), we used data collected within a live enhanced surveillance questionnaire (ESQ). The ESQ is currently used to support initial case management by collecting data on case demographics (age, sex, ethnicity), previous testing, history of pica or learning difficulties, occupational status/school attendance and hobbies, home ownership and likely sources of lead exposure.

Data from both questionnaires were extracted from Select Survey on 30^th^ September 2022.

### Case definition for inclusion in this study

The case definition for inclusion in this study was:

Residents of England, aged <16 years at the time of the first elevated BLC, reported to UKHSA HPTs between 1st January 2014 and 30th September 2022 with a BLC reportable for public health action (≥0.48μmol/L before 5th July 2021 and ≥0.24μmol/L from 5th July 2021) where BLC is known, with a completed lookback questionnaire or with a completed ESQ. All data processing was conducted in R (version 4.2.2) and MS Excel.

### Data deduplication and cleaning

For both datasets, a unique identifier was created using case first name, last name and date of birth. Duplicate records on unique ID were then manually reviewed in Microsoft Excel to ensure only unique cases were retained. Data were cleaned to meet the case definition as above.

### Data management and recoding of variables

Data from the ESQ and lookback questionnaires were merged to form one summary dataset. Questions unique to the audit questionnaire and ESQ were summarised separately.

Any free text responses were manually reviewed and re-coded to pre-existing categories used in the questionnaires or added as a new category if not already accounted for. Responses to lead exposure through drinking water and/or lead pipes were combined to form a single variable ‘drinking water’, reflecting exposure through either source.

A deprivation variable was created for each case using an online tool [15] which matched postcodes to the Office for National Statistics (ONS) [16] 2019 indices of multiple deprivation (IMD) decile in Microsoft Excel. IMD ranks small geographical areas in England from most deprived to least deprived in 10 equal groups based on different domains such as income deprivation, education deprivation, employment deprivation and crime [17].

### Data analysis

Categorical variables of interest were simplified and summarised as numbers and/or percentages of the total number of cases or subset of cases, where applicable. Continuous variables (age and BLC) were summarised by calculating median and inter quartile range. Any associations between cases and IMD decile were tested using appropriate regression techniques.

#### Qualitative data

A thematic analysis was conducted on the free-text question: “Were there any specific problems or issues that arose during the investigation and management of this case? e.g., was it difficult to engage with social services/environmental health/other stakeholders? Were there difficulties in isolating the source of poisoning or remediation, etc.?”

The qualitative analysis was conducted by the lead author (female, Master’s in Public Health and UK-Field Epidemiology Training Programme Fellow at time of research). The lead author had no prior relationship with questionnaire respondents. Questionnaire respondents were told the purpose of the exercise was to complete an audit for cases of lead poisoning in children from 2014.

The lead author broadly followed the 6 steps of qualitative analysis as outlined by Clarke and Braun, 2013 [18]: familiarisation of data, generating codes, searching for themes, reviewing themes, defining and naming themes and writing up results. For this study, free-text responses were captured in Microsoft word. Key parts of each free-text response were highlighted and assigned a code. Codes were then assigned to themes and sub-themes in Microsoft Excel. Results were summarised by theme with a free-text response selected per theme/sub-theme to provide an example. Obvious spelling errors within example quotes were corrected and abbreviations expanded. Sub themes were tabulated within main themes and presented.

All percentages presented were rounded to the nearest whole number.

### Ethics statement

No ethics approval was required for this study. All data were collected within statutory approvals granted to UKHSA for public health disease surveillance and control. Information was held securely and in accordance with the Data Protection Act 2018, General Data Protection Regulations (GDPR) and Caldicott guidelines.

## Results

### Data cleaning and deduplication

Between 1^st^ January 2014 and 30^th^ September 2022, 521 children aged <16 years and resident in England were reported to HPTs for public health investigation for lead exposure. Post data deduplication and cleaning, 340 cases (65%), had either a completed lookback questionnaire or ESQ completed and met the inclusion criteria for this study. For cases eligible for a lookback questionnaire, 241 (80%) had one completed and for cases eligible for an ESQ, 99 (45%) had one completed.

### Source of case notification and case demographics

Most cases were reported to HPTs by LEICSS (65%) followed by a paediatrician (16%, Table 1). HPTs also received case notifications from other sources such as direct reporting from non LEICSS laboratories, hospitals, and general practitioners (Table 1).

**Table 1:** Source of notification and demographics of cases (n = 340).

Variable	N (%)
Source of notification to HPT^a^	
Lead Exposure in Children Surveillance System (LEICSS)	221 (65%)
Paediatrician	53 (16%)
Laboratory	28 (8%)
Hospital	8 (2%)
General Practitioner	4 (1%)
Environmental Health Officer	2 (1%)
Parent	1 (<1%)
Notification of Infectious Diseases (NOIDs)	1 (<1%)
Clinical Toxicologist	1 (<1%)
Missing	26 (8%)
Age group (Years)	
<1	40 (12%)
1–4	180 (53%)
5–11	109 (32%)
12–15	10 (3%)
Missing	1 (<1%)
Age (Years)	3 (2; 6)^b^
Sex	
Male	235 (69%)
Female	97 (29%)
Missing	8 (2%)
Blood lead concentration (μmol/L)	0.65 (0.50; 1.03)^c^
Ethnicity	
White British	53 (16%)
Any Other	21 (6%)
Asian Pakistani	15 (4%)
Asian Other	11 (3%)
Black African/Black Caribbean	10 (3%)
Asian Indian	9 (3%)
White Other	9 (3%)
Mixed	5 (1%)
Missing	207 (61%)

^a^Percentages do not add up to 100% as multiple responses could be entered for this question

^b^Median (lower quartile; upper quartile). Excludes cases with missing age (n = 1, <1%). The minimum BLC in our sample was 0.24μmol/L and the maximum was 17.59μmol/L.

^c^Median (lower quartile; upper quartile). Excludes cases with missing blood lead concentration (n = 75, 22%)

**Fig 1:**
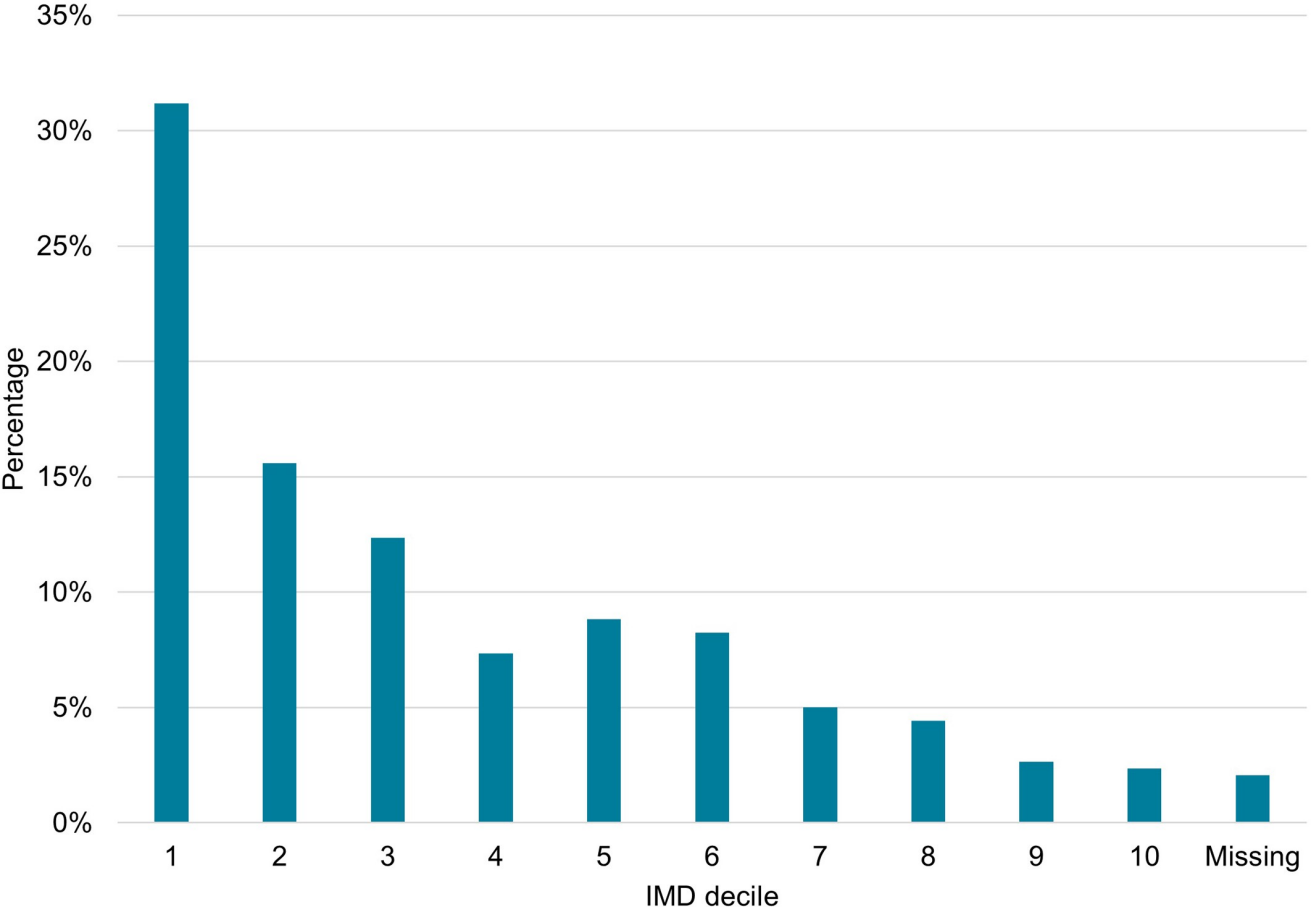
Percentage of cases by indices of multiple deprivation (IMD). Percentages reported are of the combined dataset (n = 340). IMD 1 = Most deprived decile, IMD 10 = Least deprived decile. P-value for trend (<0.001).

Fig 1 shows the distribution of cases by IMD decile. There was a general decrease in the percentage of cases with increasing IMD decile, with most cases (n = 106, 31%) categorised as IMD decile 1 (most deprived decile) and 59% of cases (n = 201) within the most 3 deprived deciles (IMD deciles 1 to 3, Fig 1). We found an exponential decreasing trend in cases by IMD decile (p <0.001).

Case age ranged from 0 to 15 years with most cases aged 1–4 years (53%, Table 2). The median age for cases was 3 years. Cases were predominantly male (69%). BLC for cases ranged from 0.24μm/L to 17.59μm/L and the median BLC for cases was 0.65μm/L (lower quartile 0.50; upper quartile 1.03) (Table 1). Ethnicity information was largely missing (61%, Table 1) but where captured, predominantly White British (16%).

**Table 2:** Likely setting and source of lead exposure, and risk factors for lead exposure in cases.

Variable	N (%)
Setting in which lead exposure likely occurred^a^	
Domestic	221 (92%)
School	3 (1%)
Hostel	1 (<1%)
Parental workplace	1 (<1%)
Shooting (gun) club	1 (<1%)
Missing	14 (6%)
Identification of exposure source^b^	
Yes–confirmed (likely by sampling)	38 (16%)
Yes–suspected (without sampling)	109 (45%)
Investigations ongoing	5 (2%)
No	76 (32%)
Missing	13 (5%)
Potential sources of lead exposure^a^	
Paint	146 (43%)
Soil	100 (29%)
Drinking water/lead pipes	19 (6%)
Food^c^	19 (6%)
Traditional (alternative) medicines	14 (4%)
Occupational (parental)	10 (3%)
Ceramics	5 (1%)
Cosmetics	4 (1%)
Lead weight	4 (1%)
Air gun/rifle pellets/shot put	3 (1%)
Cooking utensils	2 (1%)
Likely false positive (suspected contaminated blood sampling tubes)	2 (1%)
Maternal lead exposure during pregnancy	1 (<1%)
Other/miscellaneous^d^	50 (15%)
Missing	19 (6%)
Pica^a^	
Yes	259 (76%)
No	58 (17%)
Not known	9 (3%)
Missing	14 (4%)
Learning difficulty^e^	
Yes	59 (60%)
No	30 (30%)
Missing	10 (10%)
Home ownership^e^	
Yes	27 (27%)
No	63 (63%)
Missing	10 (10%)
Home rental^e^	
Yes	62 (63%)
No	20 (20%)
Missing	17 (17%)
Landlord type for rented homes^f^	
Private	23 (37%)
Council/Local Authority	17 (27%)
Housing Association	13 (21%)
Other	1 (2%)
Missing	8 (13%)
Home built before 1970^e^	
Yes	43 (43%)
No	18 (18%)
Don’t know	28 (28%)
Missing	10 (10%)

^a^Percentages reported are of the combined dataset (n = 340)

^b^Percentages reported are of the lookback dataset (n = 241)

^c^There were 10 entries where further specific detail was provided on the type of food and these included references to the following: spices, mixed spices, turmeric, chilli, chilli powder, coriander, imported sweets, dried chillies, dry fruits, pak choi, fish oil, noodles, rice

^d^Other/miscellaneous category includes references to the following: batteries, bike handlebar, books, brick, mortar, cement, chalk, clay, clothes, coins, crayons, dining table, door/fridge handles, dust, foam, garden spring, grass, hair clips/pins, hose pipe, house built on old car storage/wrecking yard, internal wall of house, leaves, metal bar, metal objects, paper, parental consumption of herbal remedy, pegs, pencils, plaster, plastic, playdough, puzzle pieces, remote control, roof of outhouse, sand/magic sand, solder, stones, substance sprayed on walls, toys/toy rattles, wallpaper, wood, woodchip wallpaper

^e^Percentages reported are of the ESQ dataset (n = 99)

^f^Percentages reported are of those who responded ‘Yes’ to renting their home (n = 62)

### Likely setting and sources of lead exposure

The most common setting where lead exposure occurred was within the domestic setting (92%) although other settings were identified (schools, hostel, parental workplace, and shooting (gun) club, Table 2). A specific exposure source was found in 61% of investigations, but only confirmed (likely by environmental sampling) in 16% of cases (Table 2). Despite this, the most common sources of lead exposure were paint (43%) and soil (29%), although other sources were identified which included drinking water/pipes, food (including, but not limited to spices, where further detail was provided), traditional (alternative) medicines, ceramics, cosmetics and lead weights (Table 2). Where traditional (alternative) medicine use was mentioned as a possible source of lead exposure and ethnicity information was also available (n = 10), 50% (n = 5) were of Asian ethnicity, although this should be interpreted with caution as case numbers were low. The majority of cases had pica (76%) and 60% had a learning difficulty (Table 2). Cases more often lived in rented homes (63%) with social rentals (48%) (council/local authority landlord and housing association landlords, being more common than private rentals (37%) (Table 3).

**Table 3:** Public health and clinical investigations and outcomes for cases.

Variable	N (%)
Was a site visit conducted^a^	
Yes	121 (49%)
No	110 (45%)
Missing	10 (4%)
Who conducted a site visit if one was undertaken^b^	
Local authority public health or environmental health staff	105 (87%)
PHE/UKHSA staff	43 (36%)
Water company	26 (21%)
Housing team/officer^c^	4 (3%)
Other local authority staff	4 (3%)
Clinician	3 (2%
Private surveyor	1 (1%)
Interpreter	1 (1%)
Missing	1 (1%)
Were control measures implemented^a^	
Yes	174 (71%)
No	42 (17%)
Missing	25 (10%)
Type of control measures implemented^d^	
Advice	125 (72%)
Alteration to home environment	65 (37%)
Rehoming^e^	13 (8%)
Other^f^	9 (5%)
Missing	2 (1%)
Clinical investigations/care^a^^,g^	
Paediatric care/paediatric outpatient care	152 (63%)
No clinical care received	25 (10%)
Repeat blood tests	19 (8%)
Treatment^h^	19 (8%)
GP care	14 (6%)
Hospitalisation	13 (5%)
Chelation therapy	8 (3%)
Referral to National Poisons Information Service (NPIS)	1 (<1%)
Accident and emergency admission	1 (<1%)
Missing	46 (19%)

^a^Percentages reported are of the lookback dataset (n = 241). Percentages do not add up to 100% as multiple responses could be entered for this question

^b^Percentages reported are of those who responded ‘Yes’ to a site visit being conducted (n = 121)

^c^Includes Local Authority housing staff and other housing staff i.e., Housing Associations and Private Landlords

^d^Percentages reported are of those who reported ‘Yes’ to a control measure being implemented (n = 174)

^e^Includes rehoming prior to PHE/UKHSA investigations commencing, consideration for rehousing and incidental rehoming whilst investigations were ongoing

^f^Other control measures refer to the following: Case removed into care, Cleaning of property, Parent only giving case bottled water, Referral to Children and Young Person’s Psychiatry, Referral to Geneticists, Stopping work at a particular site (presumably parent/guardian), Testing of toy

^g^Percentages do not add up to 100% as multiple responses could be entered for this question.

^h^Type of treatment not specified in original lookback questionnaire

### Public health and clinical investigations

A site visit was conducted for almost half of cases (49%) of which the majority (87%) were conducted by public health or environmental health staff at the local authority, with PHE/UKHSA staff attending 36% of the time (Table 3). Other stakeholders that took part in site visits included water companies, housing teams, other local authority staff, clinicians, private surveyors, and interpreters (Table 3).

Control measures were undertaken for 71% of cases of which the most commonly recorded was advice (72%) followed by alteration to the home environment (37%, Table 3). Rehoming was mentioned in 8% of cases. Where known, cases tended to be under paediatric/paediatric outpatient care (63%, Table 3). A small proportion of cases were hospitalised (5%), received chelation therapy (3%) or were admitted to an accident and emergency department (1%).

### Problems experienced during case investigations

There were 123 free-text responses out of total of 241 records (51%) entered for the question “Were there any specific problems or issues that arose during the investigation and management of this case?” in the lookback questionnaire. Of these, 8 (3%) explicitly stated no problem was experienced during investigations whilst 14 (6%) were free-text entries that were deemed unrelated to the question or did not fall into a theme.

The thematic analysis highlighted poor engagement by stakeholders or delays in response relating to case investigations, particularly relating to the parent/guardian (38%) and the local authority (15%) as the most frequently experienced problem during case management by HPTs (Table 4). This theme was different to that of contacting stakeholders (9%) which reflected difficulty in getting in touch with stakeholders specifically. Difficulty in agreeing or having clarity on roles and responsibilities was the next most frequently experienced problem experienced (13%). Specific examples given related to the following: refusal of testing possible lead exposure sources (including water) by local authority/environmental health, refusal of carrying out repeat blood tests by clinicians, responsibility for carrying out remedial work, agreeing on who should notify the case’s family of results, confusion around who needs to complete the questionnaire (presumably ESQ) with family (environmental health officer vs clinician) and who was responsible for continued enforcement of actions and case follow-up. Additional resource was required during case investigation. This involved the need for an interpreter, funding to conduct remedial work, and the need for additional expertise to support case management.

**Table 4:** Problems experienced during case investigation.

Theme/Sub-theme	N (%)^a^	Example free text quote^b^
Stakeholder engagement/response	53 (38%)	
Parent/guardian	20 (38%)	“Initially difficult to engage mother in follow up of children and getting blood tests”
Local authority	8 (15%)	“Took some time to involve the Environmental Health Officers for that area”
COVID-19	5 (9%)	“Delay in ongoing management due to COVID-19 pandemic”
Clinician	4 (8%)	“Delays in obtaining further information from reporting paediatrician”
Landlord	4 (8%)	“Delay to home visit while waiting for landlord to be available and give consent”
Notification	3 (6%)	“Delayed notification of result to HPT”
Housing association	2 (4%)	“Delays with engaging housing association regarding redecoration”
HPT	2 (4%)	“HPT capacity was limited so there was a delay to follow up during December 2020”
Informing case of results	1 (2%)	“Delay in case being informed of results causing delay in initiation of public health actions”
Laboratory	1 (2%)	“Very late lab result”
Unspecified	3 (6%)	“Difficulties with access”
Roles and responsibilities	19 (13%)	"Local authority reported they do not complete water testing anymore…” “Delay in ascertaining who should report results to family”
Additional resource	14 (10%)	
Interpreter	8 (57%)	“Interpreter assisted with communicating with family”
Funding	4 (29%)	“Family negotiated disability facilities grant…for remedial works”
Expertise	2 (14%)	“Education of the Local Authority Housing Officer about lead poisoning was required”
Contacting stakeholders	13 (9%)	
Clinical carer	7 (54%)	“Reaching the doctors involved”
Parent/guardian	4 (31%)	“Difficulty contacting family to arrange repeat tests (were on holiday, but also did not attend appointments and failed to collect blood forms)”
Landlord	1 (8%)	“Challenges informing landlord of potential lead in paint, therefore challenges in collecting paint samples”
Local authority	1 (8%)	“Difficulty in getting in contact with housing/repairs colleagues at (…) but did eventually”
Source identification	13 (9%)	“Nil significant risk factors identified”
Continuity	11 (8%)	
Clinical carer	5 (45%)	"Case’s paediatrician changed, and HPT were unable to reach the new doctor for some time”
Residence	5 (45%)	“Family moved residence during investigation…”
HPT case manager	1 (9%)	“Also due to nature of the way we work in the duty team this case was picked up by a number of different people…”
Safeguarding	10 (7%)	“Some general safeguarding concerns…”
Information errors^c^	6 (4%)	“Likely false positive result from the blood bottle used to collect the sample”
Parental worry of implications of investigation	2 (1%)	“Site visit planned by Environmental Health Officers but parent did not return messages to arrange time (concern over housing association’s reaction?)”
Total	141 (100%)	

^a^Percentages reported for main themes are of the total number of responses that were assigned a theme (n = 141). Percentages reported for sub-themes are of the total number of responses for their corresponding main theme

^b^Obvious spelling errors corrected by lead author and abbreviations expanded for publication purposes

^c^Includes false positive results (n = 3) and misspelt name/unknown laboratory details (n = 1)

## Discussion

This was the first detailed exploration of recorded settings and sources of lead exposure, public health investigations and outcomes for children with an elevated BLC in England.

### Case demographics

Demographics of cases included in this study were consistent with previously published findings [19–23] with cases predominantly being male, aged between 1–4 years old and having pica or a learning difficulty. The median BLC of cases included in our study (0.65μm/L) was higher than the BLC of laboratory detected cases in 2022 (0.37μm/L) as described in the most recent LEICSS annual report [24]. This was to be expected as the majority of the cases included in our study (71%) were those notified before the lowering of the public health intervention BLC in England on 5^th^ July 2021 [7].

### Household tenure

Sixty-three percent of cases lived in homes that were rented, of which most, were social rentals (council/local authority and housing association, 48%). The proportion of rentals in our cohort was greater than the national average. The English Housing Survey 2021–2022 highlighted 36% of households were rented in England during this time period [25]. Both the proportion of social (48%) and private rentals (37%) in our cohort were greater than the national average (17% and 19% respectively) [25]. The rental sector overall, including social and private landlords, should be key targets for public health messaging and engagement regarding lead exposure and risks of lead exposure to enable case investigation and removal of sources, particularly as engagement, contact and worry of reaction of landlords/housing authorities were identified in the thematic analysis of problems occurring during case investigation. Additionally, the private rental sector in England tends to be poorly managed and with lower standards [26]. Although originally intended to provide short-term, flexible housing for mobile workers, it has become the dominant housing tenure for poorer young adults and their children. In 2017/18, one in three children in poverty lived in private renting [26]. However, as approximately one-third of our cohort lived in owner-occupied homes, it is important to consider the needs of this group too, particularly as the ability to undertake remedial action may be further limited due to lack of access to funding for this group.

### Ethnicity and deprivation

Previous studies in the UK have shown children with an Asian ethnic background had higher BLC than children with European ethnicity [27], whilst data from the United States (US) has shown non-Hispanic Black children have higher blood lead levels than non-Hispanic White children [28]. Unfortunately, ethnicity data were largely missing in our cohort hence it is not possible to draw any valid conclusions on the ethnic makeup of the cases managed by HPTs in England.

Cases tended to live in the most deprived areas of England based on IMD decile. This could be a result of ascertainment bias as we may expect more cases to live in deprived areas. Deprived areas are more likely to have older, poorly maintained housing. Consequently, there is a greater exposure risk from leaded paint or lead pipes, with poorer conditions making these lead sources more accessible. Additionally, those living in more deprived areas, are likely to experience health deprivation and therefore, seek health and support services, and consequently be more likely to have elevated BLC detected through clinical investigations. The association between lead exposure and social deprivation has also been seen in other populations [29, 30]. However, some studies have not identified a relationship between IMD quintiles and BLC [31].

In England, there is a clear link between ethnicity and deprivation. In 2019, those with Asian ethnicity were more likely to live in the most deprived 10% of neighbourhoods in England (15.7%) followed by Black ethnicity (15.2%) based on IMD [32]. Having more complete and better quality ethnicity data for cases of elevated blood lead in England could support with identifying ethnic groups most vulnerable to lead exposure and help target public health messaging and action to reducing health inequalities for these groups.

It is also important to consider the association between socio-economic status, demographics, culture, religion, spirituality, and ethnicity with sources of lead exposure. For example, the use of ingestible complementary and alternative medicines (CAM), which may contain lead and other metals, may vary across different populations [33]. In our study, traditional (alternative) medicine was mentioned as a possible source in a small number of cases, however, the paucity of ethnicity data in our cases meant we were unable to explore associations between ethnicity and sources of lead.

### Setting and sources of exposure

In this study, lead exposure occurred almost exclusively in the domestic setting (92%). However, other settings where lead exposure may have occurred were also identified such as schools, a hostel, the parental workplace, and a shooting (gun) club.

Although the use of leaded products such as paint and water pipes in the UK has been prohibited [8], old lead containing products may still be in circulation and the data from this study indicate that lead paint (43% of cases) or leaded pipes (less commonly) are still key exposure sources in the UK. In our study, case investigations found sources of lead for 61% of cases. For the rest, a sizable proportion did not have sources confirmed or suspected, and therefore, the significance of lesser-known sources of lead should not be dismissed when interpreting this data and during case investigations. For example, a recent study of lead concentrations in a range of environmental samples from households in Georgia found that along with paint, soil and dust, spice samples had some of the highest lead concentrations [34]. The role of the ESQ is designed to explore all possible sources and prompt investigators to ask questions on all potential sources and activities. In this study, where asked, 43% of cases knowingly lived in homes built before 1970, whilst 28% did not know and therefore, this percentage could be underestimated. Studies in England [31] and internationally [35–37] have identified housing age as a risk factor for lead exposure or higher BLC. Published literature tends to focus on household characteristics (age, type) as a proxy for exposure to household sources. However, these studies lack a more detailed breakdown of household characteristics or review of case investigations to describe the most likely activities or sources of lead exposure. It would be beneficial for further studies to conduct case reviews to add to the body of evidence for likely sources of lead exposure.

Identification of lead exposure sources using sampling is difficult and not always done. Source confirmation should occur through environmental sampling (i.e., taking samples of paint, soil, water, food, spices, etc, from the home) or use of more sophisticated identification techniques such as Lead Isotope Ratio analysis [34, 38]. Although the source of lead was only confirmed in 16% of cases in our study, there are a number of factors that impact on whether environmental samples are taken and tested. Sampling is expensive and there is often a lack of clarity around the responsibility for funding sampling. HPTs may also take a pragmatic view regarding source confirmation. If a potential source has been identified in the initial discussion with parents, advice on remediation is given and there is subsequently a consistent fall in BLC, the additional expense of sampling would be seen as unnecessary. Often environmental sampling is only carried out if there is some doubt about the source of lead. The outcome of the investigation needs to clearly result in a reduction in BLC to ensure that the exposure source has been removed. Repeated BLC testing and environmental sampling may be needed if this is not achieved.

### Public health, clinical investigations, and outcomes

Almost half of all case investigations required a site visit and multiple stakeholders conducted site visits reflecting both the burden and multidisciplinary nature of case investigations. Advice was the most frequently mentioned control measure in this study, highlighting the importance of education and health messaging in managing cases and working with stakeholders to ensure the removal of exposure sources. Responsibility of enforcing agreed actions and continued follow-up was also highlighted as a problem in the thematic analysis.

Cases of elevated blood lead pose a burden on our National Health Service (NHS) and the provision of health care services and there is no minimum threshold for the adverse effects of lead exposure [1, 2]. Therefore, preventing initial and continued exposure to sources of lead are likely to help reduce referral of cases into secondary care. Exposure to lead can “damage the brain and nervous system, lead to slowed growth and development, learning and behaviour problems and hearing and speech problems” [39] which will mean a child is likely to need additional support throughout their life course for example from clinicians, speech and language therapists and educational support staff. In this study, most cases were under paediatric/paediatric outpatient care (63%). However, the primary reason for paediatrician referral is unknown, and it is important to also consider that the majority of cases in our study had pica or a learning difficulty, conditions which may also require paediatric care. Additionally, iron deficiency is associated with lead poisoning with both conditions sharing many of the same risk factors [40]. Although 8% of cases received ‘treatment’, due to the format of the question in its respective questionnaire, it is unclear what ‘treatment’ was received by cases, and this does not reflect a mutually exclusive category of care/treatment. Additionally, in our study, only 8% of cases had recorded repeat blood tests. Repeat blood testing should be undertaken for all cases referred to HPTs according to UKHSA’s national standard operating procedure (SOP) for case management, to ensure BLCs are below or reducing towards the intervention concentration. Due to the format of this question in its respective questionnaire, our findings are suggestive of poor data quality (as this information may not have been noted in the case’s record) or poor recall and should be interpreted with caution. Anecdotally, 88% of 48 cases managed in the North East and North Central London HPT in the last 5 years have at least one repeat blood test recorded–further suggesting the issue may be lack of recorded data rather than lack of testing. It is recommended that clinicians follow re-testing advice provided by UKHSA’s national SOP for case management–as in the absence of source identification, re-testing enables verification of source mitigation.

As well as the support needed by children for the management of symptoms and pathophysiology of lead exposure, the detrimental effects of lead exposure can impact a child’s educational outcomes and therefore have a subsequent effect on employment opportunities and in the longer term, socio-economic status. There is a strong body of evidence highlighting the relationship between lead exposure in childhood and violent crime in adulthood [41, 42].

### Problems experienced during case investigation

The problems experienced during case investigation highlight the complexity in the management of cases of elevated blood lead. Stakeholder engagement/response was the biggest challenge experienced during case investigations (38%). It would be important to consider how engagement/response of HPTs, local authority colleagues, housing authorities/landlords, parents/guardians, and laboratories could be improved. Raising awareness of the public health importance of lead exposure, and the importance of timely action to reduce detrimental health effects in children could be one way of doing so and should be considered via outreach work, networking or national campaigns/awareness raising events. Alongside this, targeted public health messaging and engagement with housing bodies, both social and private, would be beneficial, particularly as parent/guardian worry over the reaction of the housing association to further investigation of the home by public health officials was identified as a problem in the thematic analysis. Other studies have also highlighted challenges in requesting testing and mitigating lead paint exposure by landlords [43].

Difficulty in agreeing or having clarity on roles and responsibilities was also a common theme emerging from the thematic analysis. Although this may be more difficult to do amongst all stakeholders that may be involved in case management, having guidance and signposting documents regarding roles and responsibilities will help.

Some cases may live in complex and vulnerable circumstances, reflected in the identification of safeguarding issues during case investigation. In the context of children, safeguarding reflects “the action that is taken to protect the welfare of children and protect them from harm” [44]. Involving stakeholders such as social services and support services adds complexity to what is already a lengthy and involved response.

### Limitations

#### Ascertainment bias

Most cases included in this study were reported to HPTs via LEICSS. As a passive surveillance system, LEICSS is subject to ascertainment bias through regional variation in clinician testing of children for elevated blood lead and incidents and clusters impacting regional variation in case numbers [11]. Additionally, awareness of lead exposure varies across the country. This has resulted in geographical variation in reporting to LEICSS. Between 2015 and 2021, the highest proportion of LEICSS cases were from the Yorkshire and the Humber region [11].

#### Representativeness of cases included in this study

As part of the case definition for inclusion in this study, cases needed a completed lookback questionnaire or ESQ. Completion of questionnaires depended upon availability of case managers and access to records. However, after cleaning and deduplication, 80% of eligible cases had a completed lookback questionnaire, so the findings of this study are thought to be representative of cases reported to HPTs for investigation. A lower proportion of eligible cases had an ESQ (45%) and therefore, the representativeness of more recently reported cases should be considered with caution.

#### Data entry variability and interpretation errors

Data entry for a case record in HPZone was likely to be variable for each case depending on the HPT practitioner completing the record, complexities of the investigation and length of investigation. Review and interpretation of case records was likely to be subjective for person(s) completing the questionnaire depending on prior knowledge and experience of working with cases of elevated BLC. Additionally, interpretation of free-text responses for the qualitative analysis could be subjective and carry interpretation error, particularly as the researcher did not have a health protection/case management background.

#### Missing/incorrect data

Missing data was prevalent in our study. Retrospective review of case records to complete audit questionnaires could result in missing or incorrect data recall. For example, only 8% of cases in our study had recorded repeat blood tests, whilst anecdotal evidence suggests this is likely to be incorrect and a result of poor data quality. This study required review of cases which were managed over 7 years ago in some instances and required interpretation of historic case notes. Original case managers may no longer be available or remember the details of case management.

#### Deduplication

Manual review of duplicate records may have been subject to some degree of human error as case details could have been omitted. Measures were put in place to retain the most complete record, but some details may have been missed.

#### Questionnaire design

A large proportion of questions within the audit questionnaire contained ‘other please specify’ free-text answer options which made standardisation of responses difficult and required all free text responses to be manually reviewed and re-coded into pre-defined categories or new categories. Some details may have been coded incorrectly or omitted.

Some questions which enabled multiple answers to be submitted did not have mutually exclusive answer options. For example, for the question relating to clinical investigations/ care received by cases, including repeat testing, categories were not mutually exclusive, making interpretation difficult. Some questions lacked specificity. For example, a source was only confirmed for 16% (likely through sampling) and suspected for 45% of cases. However, this question did not specifically ask how a source was confirmed and would have benefitted from further specificity. Any future surveys should contain questions with mutually exclusive answers, more specific questions, and fewer free-text answer options.

#### Study design

This study was a descriptive epidemiology cohort study and therefore the authors were unable to test hypothesis and determine any statistically significant relationships between variables of further interest. Doing so could identify risk factors and areas to focus public health messaging or interventions. Any analytical studies using the dataset would need to consider limitations in data quality in exploring relationships between variables and the limits to interpretation.

#### Representativeness of study findings

Our findings may not be applicable to other countries. Sources of lead exposure may be different in low and middle income countries in comparison to England due to differences in legislation, enforcement, and availability of market controls of lead containing products. For example, a systematic review of studies of BLC in World Bank low and middle income country groupings found “major sources of lead exposure were informal lead acid battery, recycling and manufacture, metal mining and processing, electronic waste, and the use of lead as a food adulterant, primarily in spices” [45]. The sources found in our study did not consider these activities.

## Conclusions

This study found that children with an elevated BLC in England were most commonly exposed to lead in the domestic setting with exposure to paint and soil accounting for more than 70% of the cases. Whilst most children in this study had pica or a learning difficulty, socio-economic factors may also be contributing to their exposure risk. The majority of children in this study lived in the most deprived areas of England, in rented accommodation. Routine collection of ethnicity data for cases is vital in order to identify groups more vulnerable to lead exposure in England and to target outreach and preventative work. Another target group for stakeholder engagement are social and private rental landlords in England. Engagement could be improved via educational outreach work by public health organisations and/or national campaigns on the public health importance of lead exposure and the importance of timely action to reduce detrimental health effects.

As public health and clinical investigation of cases is complex, challenging and multidisciplinary in nature, preventing lead exposure in children, identifying control measures most effective for reducing BLC and reducing the burden of case investigation in England are important next steps. Future work should build upon the findings of this study and add to the evidence base of risk factors and vulnerable groups for elevated blood lead in children in England. Studies should explore the relationship between ethnicity, deprivation, housing tenure and blood lead concentration for targeting preventive work.
